# The weight of evidence: does obesity really not matter for atrial fibrillation ablation? Lessons from CABANA

**DOI:** 10.1093/europace/euag290

**Published:** 2026-10-29

**Authors:** Pascal Defaye, Moussa Mansour, Luigi Di Biase

**Affiliations:** Department of Cardiology, University Hospital Grenoble Alpes and Grenoble Alpes University, CHU Grenoble Alpes, CS 10217, Grenoble Cedex 9, Grenoble 38043, France; Cardiac Arrhythmia Service, Massachusetts General Hospital, Harvard Medical School, Boston, MA, USA; Department of Cardiac Electrophysiology, Albert Einstein College of Medicine at Montefiore Health System, Bronx, NY, USA

**Keywords:** Obesity, Atrial fibrillation, Catheter ablation, CABANA, Comorbidity, GLP-1 receptor agonists

**This editorial refers to ‘Impact of obesity on clinical outcomes and the treatment effect of catheter ablation in atrial fibrillation: insights from CABANA’ by J. Erzeel *et al.*, https://doi.org/10.1093/europace/euag293.**

Obesity and atrial fibrillation (AF) have grown up together, locked in a bidirectional loop: Excess adiposity drives atrial remodelling through inflammation, epicardial fat infiltration, and fibrosis, while AF perpetuates the sedentary, symptomatic state that makes weight loss even harder to achieve. Catheter ablation has become the default rhythm-control strategy for symptomatic AF, but its application in obese patients remains somewhat hesitant, out of concern that outcomes will be worse. Is this really the case, and should catheter ablation be withheld from these patients until their excess weight has been addressed? This is truly an important question, since obesity is a growing epidemic in our societies and a major driver of AF. Erzeel *et al.* now offered a partial answer based on a landmark, historic randomized reference trial on AF ablation.^1^ Fifteen years after CABANA showed that ablation, compared with drug therapy, did not significantly reduce its composite primary endpoint of death, disabling stroke, serious bleeding, or cardiac arrest, though it reduced AF recurrence and cardiovascular hospitalization,^2^ this post-hoc analysis asks whether any of that treatment effect depends on body mass index (BMI).

Surprisingly, the answer is reassuring. Among 2170 CABANA participants with recorded BMI, 51% were obese (BMI ≥30 kg/m^2^),^1^ a prevalence that alone tells you obesity is no longer a comorbidity of AF; it has, in the end, become an entirely unremarkable phenotype. Obese patients were younger yet paradoxically sicker: more hypertension, diabetes, sleep apnea, and coronary disease; more persistent and long-standing persistent AF, lower left ventricular ejection fraction, and worse NYHA class and Canadian Cardiovascular Society AF-severity scores. Each 1 kg/m^2^ rise in BMI added roughly 1% to the hazard of cardiovascular hospitalization or death, and patients with class-II obesity or higher (BMI ≥35 kg/m^2^, 22% of the cohort) carried a 17% higher risk of that composite endpoint than thinner patients. Yet when the authors asked the sharper question, whether obesity changes what ablation does relative to drug therapy, the answer, across every endpoint tested, was no. Composite outcome, cardiovascular hospitalization, functional class, AF-severity score, and AF recurrence itself: every interaction term was non-significant, whether obesity was defined at 30 or 35 kg/m^2^, or analysed as a continuous variable.

This is, in one sense, the finding everyone hoped for: ablation should not be rationed by BMI. But it sits uneasily against a substantial observational literature in which obesity behaves as an independent, dose-dependent predictor of AF recurrence after ablation. A large European multicenter cohort found 12-month relapse rates climbing from 35% in normal-weight patients to 48% in the morbidly obese, with BMI an independent predictor of recurrence.^3^ A large US series reported a comparable decrease in long-term freedom from arrhythmia with rising BMI class,^4^ and a dose-response meta-analysis of 26 cohorts and more than 26 000 patients calculated that each 5 kg/m^2^ rise in BMI increased recurrence risk by 15%.^5^ Contemporary national registries sit in between. Dutch^6^ and Korean^7^ pulmonary-vein-isolation cohorts show that obese patients gain quality of life and procedural safety similar to non-obese patients, though some still report a residual gradient in arrhythmia-free survival by BMI category. *Table 1* summarizes this apparent divide. So, where does the truth lie—and should ablation really be offered regardless of BMI, with the same expectation of success?

**Table 1:** The CABANA obesity analysis in context: randomized, propensity-matched, and observational evidence on obesity and outcomes after catheter ablation for atrial fibrillation

Source	Study design	Population	Key finding on obesity/BMI and ablation outcome
INDEX STUDY—THE CABANA OBESITY ANALYSIS UNDER DISCUSSION IN THIS EDITORIAL			
Erzeel *et al.*, 2026^1^	Randomized controlled trial exploratory, non-prespecified post-hoc subgroup/interaction analysis of CABANA	*n* = 2170	No treatment-by-obesity interaction for the composite endpoint, CV hospitalization, functional status, or AF recurrence; obese patients nonetheless had worse absolute outcomes and function.
RANDOMIZED-TRIAL EVIDENCE (COMPARATORS)			
Chen *et al.*, 2025^8^	Randomized controlled trial post-hoc analysis (same CABANA dataset)	*n* = 2204	Ablation showed the greatest relative benefit on hard outcomes in patients with the highest comorbidity burden.
Sclafani *et al.* (LOSE-AF), 2026^9^	Randomized controlled trial, prospective, parallel-group	*n* = 118, older, persistent AF	A genuine 9.7% weight loss did not improve AF symptom severity, burden, or need for further rhythm control.
OBSERVATIONAL EVIDENCE—COHORTS, REGISTRIES, AND PROPENSITY-MATCHED ANALYSES (NOT RANDOMIZED)			
Providência *et al.*, 2019^3^	Observational cohort retrospective, multicenter, multivariable-adjusted (not randomized)	*n* = 2497	12-month recurrence rose from 35% (normal weight) to 48% (morbid obesity); BMI an independent predictor of recurrence (HR 1.01 per kg/m^2^).
Winkle *et al.*, 2017^4^	Observational cohort retrospective, single-center	Large US cohort	Rising BMI class associated with reduced long-term freedom from arrhythmia and more repeat procedures.
Vermeer *et al.*, 2024^6^	Observational prospective national registry	Netherlands, real-world	Comparable post-PVI quality-of-life gains across BMI categories.
Ahn *et al.*, 2024^7^	Observational retrospective national registry	Korea, cryoballoon	Similar procedural safety across BMI categories; recurrence gradient attenuated vs. radiofrequency series.
Venier *et al.*, 2026^10^	Observational retrospective cohort, 1:1 propensity-score-matched (not randomized)	*n* = 6700, obese	GLP-1 receptor agonist use associated with lower AF recurrence, permanent AF progression, CV hospitalization and mortality.
Pathak *et al.* (LEGACY), 2015^11^	Observational prospective cohort, non-randomized structured-program comparison	*n* = 355	≥10% weight loss associated with 86% vs. 40% long-term arrhythmia-free survival.
META–ANALYTIC EVIDENCE			
Liu *et al.*, 2022^5^	Meta-analysis dose-response, pooling 26 observational cohort studies	26 studies, *n* = 26 450	Linear dose-response relationship: +15% AF recurrence risk per 5 kg/m^2^ rise in BMI (95% CI 1.08–1.22).

BMI, body mass index; CV, cardiovascular; HR, hazard ratio; PVI, pulmonary vein isolation.

We would argue CABANA is not wrong, but rather that it poses a different question, with a different metric. Based on CABANA, one could be tempted to perform AF ablation regardless of BMI stratum, but this message calls for nuance, and prudence should prevail. Several features of the trial plausibly mute a signal that is real elsewhere. CABANA enrolled patients between 2009 and 2016, at a time when radiofrequency was the only energy source available, with substantial operator-dependent and also inter-centre variability in lesion delivery, and none of the technology in routine use today. Radiofrequency ablation was performed without contact-force sensing; mapping systems were of an early generation, cryoablation did not yet exist, let alone pulsed-field ablation; and energy sources now known to carry substantially lower morbidity and much shorter procedure times. These considerations matter all the more when applied to obese patients, who carry a higher risk of complications. Ultrasound-guided venous access, now the systematic standard of care, is likewise particularly important in this population and was not widely used at that time, particularly for obese patients. In CABANA, recurrence rates were high across every BMI stratum, compressing the range in which a gradient could emerge; repeat ablation was permitted during follow-up and likely diluted late recurrence signals; and the class-II-obesity subgroup, at only 476 patients, was underpowered for a formal interaction test in what its own authors rightly call an exploratory, non-prespecified analysis. Left atrial volume, arguably the best indicator of the atrial cardiomyopathy associated with obesity, and an established determinant of recurrence after pulmonary-vein isolation, was simply unavailable in this sub-study, so obese patients with remodelled, dilated atria may have been indistinguishable, in this analysis, from obese patients who were not.^12^

None of this diminishes the paper’s central message, which deserves to be heard clearly: obesity is not, on this evidence, a reason to withhold catheter ablation. If anything, this finding reinforces a companion analysis from the same trial, which found that patients with the highest comorbidity burden derive the greatest—not the smallest—relative benefit from ablation on hard clinical outcomes.^8^ Quite simply, catheter ablation must go hand in hand with obesity-management measures that need to be implemented just as systematically.

The 2024 ESC guidelines^13^ already made this official policy, elevating comorbidity and risk-factor management to the ‘C’ of the new AF-CARE pathway, a Class I recommendation with an explicit target of at least 10% body-weight reduction. Yet a contemporary EHRA survey^14^ found that only 23.5% of AF patients with elevated BMI were ever referred for formal dietary advice, a gap Erzeel *et al.* rightly invoke to contextualize their own null interaction. We ablate triggers with ever-increasing precision while leaving the organ that generates the substrate, adipose tissue itself, almost untouched in routine care. Structured programs combining weight loss with comprehensive risk-factor management, rather than pursuing either in isolation, have shown long-term arrhythmia-free survival rise from under 40% to over 85%,^11^ a magnitude of benefit no ablation strategy has matched on its own.

Pharmacology may finally be closing part of that gap. In a real-world cohort of obese patients undergoing AF ablation, GLP-1 receptor agonist use was associated with significantly lower AF recurrence, slower progression to permanent AF, fewer cardiovascular hospitalizations, and lower mortality,^10^ consistent with meta-analytic evidence that semaglutide reduces incident AF more broadly.^15^ Proposed mechanisms extend well beyond weight loss itself, including preferential reduction of epicardial adipose tissue, systemic anti-inflammatory effects, and favourable autonomic and haemodynamic remodelling. It is tempting to declare the obesity-ablation problem solved by injection of GLP-1-agonists. The LOSE-AF trial^9^ should temper that enthusiasm: in older patients with persistent AF randomized to an intensive, effective weight-loss program that achieved a genuine 9.7% body-weight reduction, AF symptom severity did not improve compared with usual care, nor did AF burden, cardiac remodelling, or the need for further rhythm-control procedures. Weight lost late, in an already remodelled, persistent-AF atrium, may simply arrive after the point of no return, precisely the phenotype that predominates among obese patients in CABANA. Timing, patient selection, and integration with rhythm control, not the kilograms alone, appear decisive.

CABANA and the literature it seems to contradict are not, in the end, in conflict; they describe two different moments of the same disease. By the time an obese patient with AF reaches the ablation lab, the procedure can only do what it is built to do—eliminate the triggers located within the pulmonary veins and, at times, in extra-venous atrial tissue—and it does so indiscriminately, regardless of what the scale reads. What it cannot do is erase years of atrial fibrosis and comorbidity accumulation that occurred before the patient was referred to us. To move the field forward, what is needed is a genuine trial in obese AF patients, randomizing ablation alone against ablation integrated with structured management of obesity—weight and comorbidities—pharmacologically supported by GLP-1 receptor agonists, with left atrial imaging and contemporary ablation technology built into the study design from the outset. Until such evidence exists, our obese patients with AF should leave the ablation laboratory with a prescription for their rhythm and an equally firm one for their weight, with drastic targets.
